# Medical Society of the State of New York

**Published:** 1873-02

**Authors:** F. C. Curtis


### Medical Society of the State of New York.

SIXTY SEVENTH ANNUAL MEETING.

Reported by F. C. Curtis, M. D.

First Day's Proceedings.

The Medical Society of the State of New York met at Albany, February 4th, 1873, in the public hall, Perry building. The meeting was called to order by the President, Dr. C. R. Agnew, of New York. Prayer by the Rev. Dr. Clark, after which the President read his inaugural address.

On motion of Dr. Vander Poel, a committee of three was appointed by the President on the President's address.

The President then announced the following committees:

On Credentials—Drs. W. H. Craig, of Albany; J. G. Orton, of Binghamton, P. V. S. Pruyn, of Kinderhook.

Drs. Orton and Pruyn not being present, Drs. A. L. Saunders, of Brookfield, Madison County, and C. E. Rider, of Rochester, were appointed in their place.

Business Committee—Drs. Ellsworth Eliot, of New York; J. V. Kendall, of Baldwinsville; G. H. Hubbard, of Lansingburgh.

Committee on Arrangements and Receptions—Drs. J. V. P. Quackenbush and W. H. Bailey. of Albany; Darwin Colvin, of Clyde, Wayne County.

Committee on Medical Ethics—Drs. Thomas Hun, of Albany; E. R. Squibb, of Brooklyn; D. B. St. John Roosa, of New York.

On motion of Dr. Frazier, of Camden, Oneida County, a committee of three were appointed by the president, to invite the Governor and such members of the Legislature as belong to the regular Medical profession.

Committee—Drs. Robert Frazier, of Camden, Oneida county; Lewis Post, of Lodi, Seneca county; Thompson Burton, of Fultonville, Montgomery county.

The President then introduced Dr. G. W. Barr, of Titusville, delegate from the Pennsylvania Medical Society. Dr. Barr was formerly from this State and served in the late war, under a commission tendered him by Dr. Vander Poel, then Surgeon General of New York.

Dr. Eliot, chairman of the Business committee, announced a paper by Dr. George Burr, of Binghamton, to be read by Dr. W. C. Wey, of Elmira, Dr. Burr being absent. Subject of paper; A case of occlusion of the femoral artery from fracture of the femur, followed by mortification and amputation. It was referred to the committee on publication. The business committee also announced a paper by E. H. Bridges of Ogdensburg. Subject: "Disease of the left ovary resulting in fatal hemorrhage." Referred to publishing committee.

The following papers were read by title: "Obituary Notice of Dr. Darius Clark," by Dr. B. F. Sherman; "Hernia," by Dr. J. H. Pooley, of Yonkers; "The Effects of Railroad Travel on the Health of Women," by Eli Van De Warker; "Idiopathic Peritonitis," by Dr. Joseph Lewi, of Albany; all of which were referred to the committee on publication.

Dr. D. B. St. John Roosa, of New York, read a paper, entitled "History of of the progress of Otology," which was discussed by Dr. Knapp, of New York. It was referred to the publishing committee.

Dr. Wm. B. Alley, of Nunda, read a paper on "The Ultimate Result of Nerve Injuries in Gunshot Wounds," which was discussed by the members of the society.

The business committee inquired what disposition the society would direct in regard to papers of invited members.

Dr. Roosa offered the following:

Resolved, That the committee be requested to consider papers that may be presented by invited members of the society, and cause them to be read if thought expedient.

The president announced the following committee on president's address: Drs. Vander Poel, John P. Gray, E. Krackowizer.

The Society then adjourned until 3 P. M.

### AFTERNOON SESSION.

Dr. Agnew, President in the chair.

Dr. J. W. S. Gouley, of New York, read a paper on "Perineal Lithotomy," which was discussed by Dr. Krackowizer, of New York, and Dr. Hutchinson, of Brooklyn.

The President announced the following committee on nominations:

First district, Dr. J. C. Hutchison; second, Dr. J. Foster Jenkins; third, Dr. H. B. Whiton; fourth, Dr. Alexander Ayres; fifth, Dr. Alonzo Churchill; sixth, Dr. William C. Wey; seventh, Dr. Caleb Green; eighth, Dr. H. W. Dean.

Dr. Vander Poel read his report in regard to changing the time of meeting of this Society.

On motion of Dr. Eliot, the report was received, and the discussion postponed till the evening session.

Dr. Thomas Addis Emmet read a paper on "Laceration of the Perineum, involving the Sphincter, and Operation for Securing Union of the Muscle." It was referred to the committee of publication.

Dr. E. H. Parker remarked upon a case of "Dislocation of the Tarsus from the Astragulus," exhibiting the specimen.

Dr. E. M Moore, of Rochester, read a paper on "Intra-Capsular Fracture," illustrated by two cases. It was discussed by Dr. Gurdon Buck, Dr. Douglas and Dr. A. N. Bell.

Dr. E. M. Hunt, delegate from the New Jersey State Medical Society, was then introduced to the society and responded gracefully to his introduction.

The Secretary, Dr. W. H. Bailey, read the following: The Medical Society of the county of Albany requests the pleasure of meeting the members of the State Society, delegates, permanent, honorary and invited members, in this room (Perry hall) to-morrow, Wednesday evening, at 9 o'clock, immediately after the President's address. On motion the invitation was accepted.

Dr. Eliot, from business committee, read the following papers by title, and they were referred to the publishing committee:

"Case of Fatty Tumors of the Abdomen," by James S. Bailey, M. D.

"Senile Hypertrophy of the Prostrate Gland," by James S. Bailey, M D.

"Unusual Case of Inflammation of the Urinary Organs," by S. T. Clark, M. D.

On motion, the society adjourned to meet at eight in the evening.

EVENING SESSION.

The Society came together at 8 o'clock P. M., Dr. B. F. Sherman, Vice-President, in the chair.

Dr. E. R. Squibb, of Brooklyn, chairman of the committee on pharmacopœia, read his report. The report, on motion, was accepted.

A paper entitled "Dropsy after Scarlatina" by Dr. George Douglas, was read by title and referred to publication committee.

The report of the committee on changing the time of meeting of the Medical Society of the State of New York was then taken up in accordance with the recommendation of the business committee. Various motions and amendments were submitted, and while the matter was under discussion, a motion to adjourn to meet at nine o'clock on Wednesday morning was made, seconded and unanimously adopted.

---

SECOND DAY'S PROCEEDINGS.—MORNING SESSION.

The Society met, agreeable to adjournment, at nine o'clock, Dr. Agnew, the President, in the chair. No clergyman being present, the President opened the meeting with prayer. The Secretary read the minutes of Tuesday's sessions. On motion they were adopted.

Dr. Oliver White, of New York, chairman of the committee on By-Laws, through the Secretary, made his report. The report was received.

The Secretary announced that he had received letters from several distinguished physicians regretting their inability to be present at this meeting. Among others he mentioned Dr. Henry I. Bowditch, of Boston; Dr. C. E. Brown-Sequard, of New York; Dr. William Brodie, of Detroit; and Dr. E. M. Snow, of Providence, Rhode Island.

Dr. F. N. Otis, of New York, read a paper on "Strictures of the Male Urethra, with Results of Operation with the Dilating Urethrotome."

Dr. Gouley, of New York, and Dr. Newman, of New York, discussed the paper.

Dr. Roosa arose to a question of privilege, making remarks and explanations regarding a paper in the Transactions of last year by Dr. Stephen Rogers, of New York.

Dr James R. Leaming, of New York, read a paper on "Plastic Exudation within the Pleura." Referred to the publishing committee.

Dr. Charles H. Porter, Treasurer, made his annual report. On motion, it was referred to the following auditing committee, appointed by the chair: Drs. J. V. Cobb, H. Corliss and P. P. Staats.

Dr. Elliot offered the following resolution:

*Resolved*, That candidates elected to permanent membership who neglect to pay the fee required by our By-Laws for one year from the date of their election, shall forfeit their rights as permanent members. Adopted.

Dr A. N. Bell read his report on the "Quarantine establishment of New York," being a report of the committee on Hygiene.

Dr. Frazier reported as follows: Your committee appointed to invite the

Governor, and medical gentlemen who are members of the Legislature, have performed that duty. Received.

Dr. A. B. Burger, of Saratoga county, presented a specimen of a diseased kidney with a description of the case. It was referred to the publishing committee.

Dr. Wm T. Lusk, of New York, read a paper on the Pathology of Labor Pains. It was referred to the publishing committee.

Dr. Gurdon Buck presented a case of reconstruction of the under lip. Referred.

Dr. Cobb, of the committee to whom was referred the Treasurer's report, reported that they had examined the same and find it to be correct.

Dr. M. H. Eddy, of Middlebury, Vermont, delegate from the Medical Society of the State of Vermont, was introduced to the Society; also, Dr. John J. H. Love, delegate from the New Jersey State Medical Society; also, Dr. S. L. F. Simpson, of Concord, New Hampshire, all of whom responded to their introductions.

Dr. Lewis A. Sayre read a paper on Diastasis of Head of Femur and Formation of Artificial Hip Joint. Referred to the publishing committee.

Dr. J. V. P. Quackenbush read a paper on "Hydrorrhœa."

Dr. E. R. Squibb reported from the committee on Ethics regarding Niagara County Medical Society. After stating the position in that county, he gave the conclusion to which the committee had arrived, as follows: "The act of disbanding the Society was null and void That, acting under the advice of this Society in January, 1871, the Medical Society of Niagara County of 1823 was again in full operation. That, although we think Dr. Clark was in error in stating that the Society dated from 1871, and not 1823, yet this statement could not render the previous proceedings invalid. We, therefore, propose the following resolution:

*Resolved*, That this Society recognize the organization in Niagara county, of which A. G. Skinner is now President, and J. L. Buckner, Secretary, as the Niagara County Medical Society, and will receive Rexford Davison and Chas. N. Palmer, delegates regularly appointed by said Society.

His report was unanimously adopted.

An obituary of Dr. P. Van Olinda, by Dr. A. Van Derveer, was read by title and referred to the publishing committee.

On motion, the Society adjourned to meet at 2.30 P. M.

AFTERNOON SESSION.

The meeting was called to order by the President, Dr. Agnew. Dr. Douglas made his report as delegate to the Connecticut State Medical Society. Dr. Corliss reported as delegate to the Maine Medical Association. Dr. Newman reported as delegate to the New Jersey State Medical Society.

Dr. Jacobi, from the committee on Foundling Asylums, made his report. Dr. Joel Foster made a minority report on the same On motion the majority report was accepted and adopted.

Dr. Kendall, of Baldwinsville, offered the following:

*Resolved*, That the By-Laws of this Society be altered so that section eleven of paragraph three shall read as follows:

¶ 3, § 11. At the annual meeting, at the close of the morning session of the first day, the members of the Society shall be organized into eight committees by Senatorial districts, as established by the law of 1836, the members present from each district constituting one committee, each of which shall elect one member; the members thus elected, with one appointed by the President as chairman, shall constitute the committee of nominations.

It was moved and seconded that it be laid on the table for the present Carried.

Dr. Storck offered the following:

*Resolved*, That the bill entitled "An act relative to the medical laws of the State of New York," passed by both houses of the Legislature last year, but vetoed by Governor Hoffman, meets with the approval of this Society.

*Resolved*, That a committee of three be appointed by the chair to take all necessary steps to secure the passage of said act by the Legislature, during its present session.

Dr. Vanderpoel, Dr. Squibb and Dr. E M. Moore discussed the resolution. On motion, it was laid on the table.

Dr. Newman offered the following:

*Resolved*, That when we adjourn, we adjourn this annual meeting to the fourth Tuesday in September, for the transaction of executive business.

*Resolved*, That after this year the annual meeting shall be held at Albany on tne fourth Tuesday of September.

*Resolved*, That the Secretary be authorized to make the amendment of the By-Laws legal, if legislation is required. After much discussion the whole subject was laid on the table.

Dr. B. L. Hovey read a report of five consecutive casses of Colles fracture. It was referred to the publication committee.

Dr. J. P. Gray exhibited to the Society micro-photographs of brain tissue

Dr H. Knapp read a paper on "Hemiopic and Sector-like Defects of the Field of Vision, and their connection with diseases of the Heart and Brain." It was referred to the business committee.

Dr. J. P. Palmer, of Victor, Ontario county, read a paper on "Spotted Fever."

Dr. Corliss read the "Biography of Dr. B. P. Staats, of Albany."

The following papers were read by title and referred to the publishing committee:

"Three cases of Abscess and Pelvic Peritonitis from Ulceration of the Appendix Vermiformis Relieved by operation," by Dr. R. B. Bontecou, of Troy; "Complete Dislocation of the Tenth Dorsal Vertebra Forwards," by Dr. Graves, of Steuben county; "On the use of Atropine in some Diseases of the Eye," by Dr. Edwin Hutchinson, of Utica—sent as a communication by the Oneida County Medical Society.

Dr. Babcock reported for the censors of the eastern district that they examined E. V. Stryker and found him well qualified, and recommended him as a proper person to receive a diploma from this Society. Adjourned.

The Society convened in the Assembly Chamber at eight o'clock to listen to the annual address by the President, Dr. C. R. Agnew. The principal subjects touched upon were the necessity of special culture for any particular study; that general culture was a necessary preliminary to special study; that an academic education was necessary to the study of medicine properly, and to train the mind to educe from general principles, practical results. He also spoke fully on the subject of sanitary arrangements in schools, illustrating the facts that bad ventilation and cramming education did more to shorten life by producing physical debility and mental imbecility than any other cause. The doctor concluded by asking all the members, on returning to their respective homes, to look well to their schools, and remedy, if possible, the evils spoken of.

Dr. James McNaughton said that he had listened with great pleasure to the able address of the President, and trusted that copies of the same would be distributed round the country. As a public teacher of fifty years standing, he could indorse the whole of the learned doctor's remarks, especially those relating to primary education, and he hoped that his own city (Albany) would profit by them. After commenting on several other subjects, he moved that a vote of thanks be accorded to the President for his address, and that a copy be distributed to each of the members. Carried.

The meeting then adjourned and proceeded to Perry hall, where, after being severally introduced to the President by Dr. Bailey, in the reception room, the members assembled in the large hall, where a sumptuous banquet had been prepared for them by the Albany County Medical Society.

Mr. W. H. Bogart "Sentinel" of the New York *World*, by request of Dr. Agnew, welcomed the gentlemen present in the name of the Albany County

Society. He said that, although the President had made the "eye" his particular study, he must have been at fault when he picked him out to extend the welcome of the County Society to the State Society, as he felt himself to be wanting in words adequate for the occasion. In welcoming tbe Society he likened it to the son's welcome to the father, for the State really was parent to the county. After dwelling on the beauties of the medical science, and alluding to embolism as the cause of Napoleon's death, explaining its meaning to be a stoppage of the heart's pulsation by clots of blood, he said that there was no such malady as embolism in his welcome, but the heart beating with all the warm impulses of friendship he bade the State Medical Soeiety welcome in the name of the County Society.

THIRD DAY'S PROCEEDINGS.—MORNING SESSION.

The Society met at 9:30 A. M. The President, Dr. Agnew, in the chair.

Prayer was offered by the Rev. Mr. Reeves. The minutes were and approved.

Dr. Vanderpoel, chairman of the committee on the President's address, read their report, and after discussion it was accepted and adopted.

Dr. Robertson, of Albany, read a paper entitled "Cases in Practice." Referred.

Dr. J. Marion Sims read a paper entitled "A case of Enucleation and removal of an Intra-Uterine Fibroid Tumor," with instruments for the same. Referred.

Dr. Wm. C. Wey read a paper entitled "Some observations concerning the Hypodermic injection of Ergot in Uterine Fibroids."

It was discussed by Drs. Sims and Squibb, and other gentlemen, and then referred to the committee on publication.

Dr. J. N. Northrop, of Albany, presented a case of congenital loss of the right arm, with remarks upon it.

Dr. Jacobi believed that it was a case of arrested developement, the reasons for which he explained in full.

The business committee read the following papers by title:

"Penetrating gun-shot wounds of cranium with recovery," by A. Van Derveer, M. D.

"Sequelae of a case of purpura hemorrhagica," by H. S. Cranall, M. D.

Dr. C. Devol presented two pathological specimens.

Dr. A. N. Bell offered the following:

*Resolved*, That the Standing Committee on Hygiene be added to, and hereafter recognized as one of the standing committees under section 111, page 20, of Organization and By-Laws of the Society, and that said committee place itself in immediate correspondence with the county societtes.

Unanimously adopted.

Dr. A. N. Bell offered the following:

*Resolved*, That the Standing Committee on Hygiene consist of seven members, one of whom shall be Dr. C. R. Agnew; the balance of the committee to be appointed by the chair. Adopted.

The President appointed the following committee on Hygiene:

Drs. A. N. Bell, S. O. Vanderpoel, H. D. Didama, H. W. Dean, John Ordronaux, Stephen Smith, C. R. Agnew.

The nominating committee reported through their Secretary, Dr. J. Foster Jenkins, as follows: The committee on nominations have the honor to report that they have unanimously agreed to recommend to the Society the following nominations to office for the ensuing year:

For President, Dr. E. M. Moore, of Rochester; Vice-President, Dr. Francis Burdick, of Johnstown; Secretary, Dr. William H. Bailey, of Albany; Treasurer, Dr. Charles H. Porter, of Albany.

The gentlemen nominated by the nominating committee were unanimously elected.

Dr. Jenkins offered a resolution, as a part of the report of the committee, that no one should be eligible for election to permanent membership until he had been a delegate for three years.

Dr. Squibb moved to amend this so as to require delegates to be present at the meetings and serve for three years. After considerable discussion the resolution was carried as amended, and the report of the committee adopted.

Dr. F. B. A. Lewis offered the following:

*Resolved*, That this society extend their sincere thanks to the officers and members of the Albany County Medical Society for their munificent entertainment and warm reception of last evening. Carried.

Dr. Agnew made a few appropriate remarks in conclusion of his services as President.

On motion of Dr. Elliot, the Society adjourned till the 1st Tuesday in Feb, 1874.

